# The roles of inflammasomes in cancer

**DOI:** 10.3389/fimmu.2023.1195572

**Published:** 2023-07-11

**Authors:** Zihan Deng, Lisen Lu, Binghui Li, Xiujuan Shi, Honglin Jin, Weidong Hu

**Affiliations:** ^1^ Department of Thoracic Surgery, ZhongNan Hospital of Wuhan University, Wuhan, Hubei, China; ^2^ College of Biomedicine and Health and College of Life Science and Technology, Huazhong Agricultural University, Wuhan, Hubei, China

**Keywords:** inflammasome, cancer, immunology, immunotherapy, nanoparticle agents

## Abstract

Inflammation is a key characteristic of all stages of tumor development, including tumor initiation, progression, malignant transformation, invasion, and metastasis. Inflammasomes are an important component of the inflammatory response and an indispensable part of the innate immune system. Inflammasomes regulate the nature of infiltrating immune cells by signaling the secretion of different cytokines and chemokines, thus regulating the anti-tumor immunity of the body. Inflammasome expression patterns vary across different tumor types and stages, playing different roles during tumor progression. The complex diversity of the inflammasomes is determined by both internal and external factors relating to tumor establishment and progression. Therefore, elucidating the specific effects of different inflammasomes in anti-tumor immunity is critical for promoting the discovery of inflammasome-targeting drugs. This review focuses on the structure, activation pathway, and identification methods of the NLRP3, NLRC4, NLRP1 and AIM2 inflammasomes. Herein, we also explore the role of inflammasomes in different cancers and their complex regulatory mechanisms, and discuss current and future directions for targeting inflammasomes in cancer therapy. A detailed knowledge of inflammasome function and regulation may lead to novel therapies that target the activation of inflammasomes as well as the discovery of new drug targets.

## 1 Introduction

The occurrence and progression of cancer is characterized by several hallmarks, including uncontrolled proliferation, autocrine growth signals, anti-apoptotic signaling, neovascularization, increased tissue invasion and metastasis, and an inflammatory environment (1). The correlation between inflammation and cancer was first discovered in the 19th century by Rudolf Virchow, who observed the infiltration of leukocytes in tumor tissue and suggested that cancer could occur at the site of chronic inflammation (2). The innate immune system can modulate cancer-associated inflammation, either by triggering the malignant development of tumors, or by providing an inflammatory microenvironment that promotes tumorigenesis (3). Inflammasomes are involved in innate immunity and mediate the release of inflammatory cytokines, trigger inflammatory cascades and promote the formation of an inflammatory microenvironment that is conducive to tumor cell growth. Therefore, a detailed study of the molecular mechanisms that govern inflammation and cancer may lead to the development of novel therapeutic approaches to cancer treatment.

An inflammasome is a kind of multiprotein complex that is assembled in cells in response to pathogen associated molecular patterns (PAMPs) and damage associated molecular patterns (DAMPs). In response to external stimulation, such as the presence of bacterial pathogens, procaspase-1 is activated and the precursors of IL-1β and IL-18 are cleaved to release the active forms of these cytokines, which result in the death of phagocytes (4, 5). Inflammasome complexes affect a variety of pathogens and their homeostatic signaling, while also modulating the host response, especially in the context of bacterial infection, autoimmune diseases, and tumors (6). Members of the nucleotide-binding domain and leucine-rich repeat containing receptor (NLR) family, NLRP1, NLRP3, NLRC4 and absent in melanoma 2 (AIM2), have been confirmed to be involved in the assembly of inflammasomes (7). Inflammasomes play an important role in the different stages of development of numerous inflammatory and tumor-like diseases in humans. They not only promote tumorigenesis and metastasis, but also exert tumor-suppressive effects, reflecting the complexity of inflammasome action during the process of tumorigenesis (8). Herein, we review the composition and activation process of different inflammasomes, and analyze the molecular mechanisms by which they may mediate tumor pathogenesis, to obtain a deeper understanding of the molecular mechanisms of tumor development. In addition, we also discuss possible therapeutic directions and potential targets for cancer therapies directed at inflammasomes. Overall, understanding the mechanisms of inflammasome formation and action in different cancers and different stages of tumor formation, development, progression, and invasion will provide new targets for clinical drugs and immunotherapies.

## 2 Overview of inflammasomes

**Figure 1:**
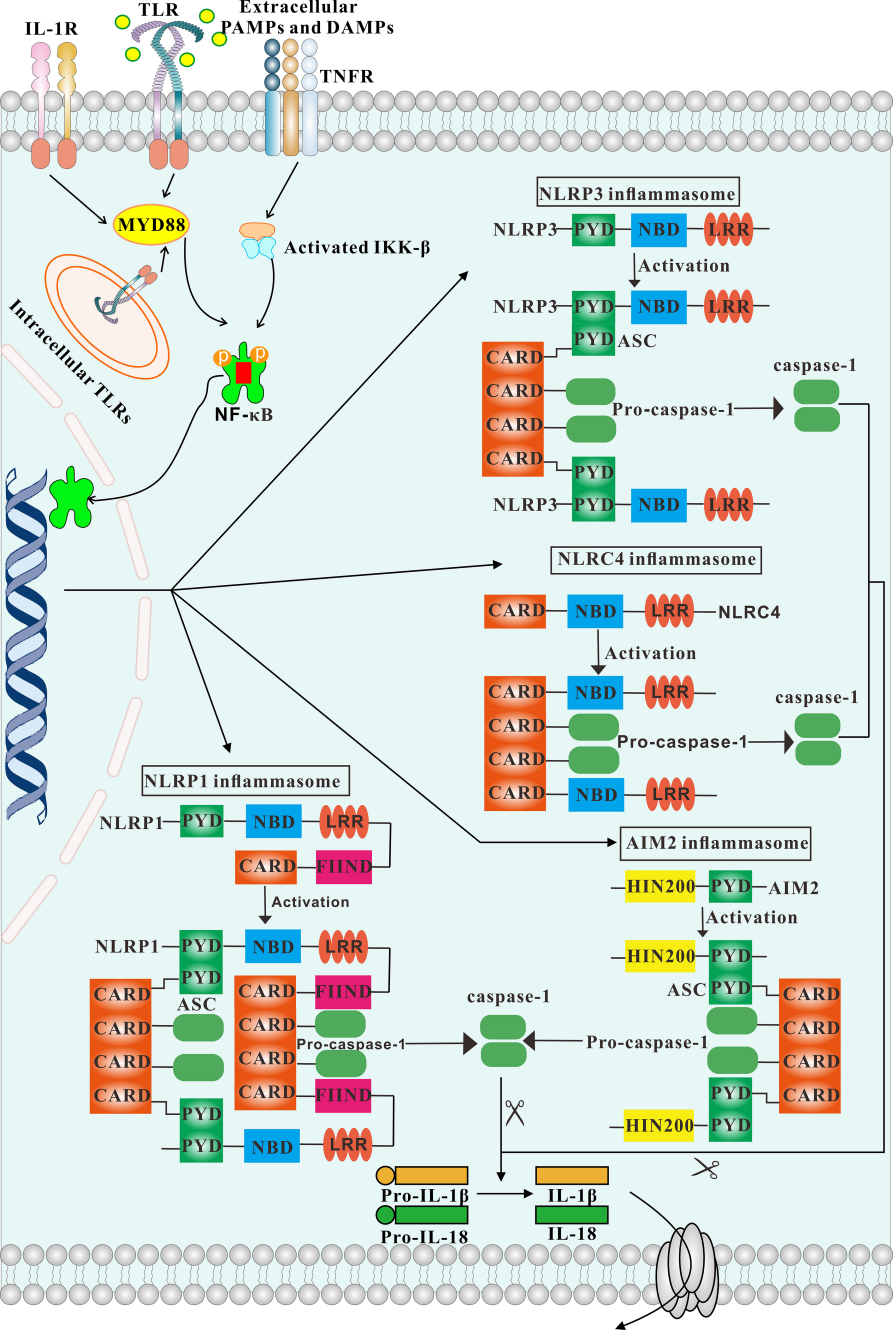
Overview of different inflammasomes. The structure and activation process of inflammasomes are depicted. Engagement of TLR, IL-1 and TNF receptors lead to activation of IKKs, which in turn results in activation of NF-κB signaling. Consequently, NF-κB activates inflammasomes. The NLRP3, AIM2 inflammasomes is strictly dependent on the adaptor ASC. In contrast, NLRP1 and NLRC4 possess a CARD domain and can recruit caspase-1 directly.

The notion of the inflammasome was first proposed by Tschopp et al. in 2002 (9). The inflammasome consists of three parts. The first is the pattern recognition receptor (PRR), which acts as an intracellular receptor. The second is the cytokine activation system, that is, ASC (apoptosis-associated speck-like protein containing a caspase recruitment domain (CARD)], which is an adaptor protein consisting of a pyrin domain (PYD) and a CARD. One end of ASC is linked to the PRR, and pro-caspase-1 is recruited through its CARD domain at the other end. Once activated, pro-caspase-1 becomes caspase-1, which is the third important component of the inflammasome. Caspase-1 can mediate the inflammatory response, induce pyroptosis and activate caspase enzymes to cause cell lysis and apoptosis (10, 11). Because the structure of adaptor protein ASC and effector protein caspase-1 are rarely changed, inflammasomes are generally classified according to the different receptor proteins, which are more varied. Inflammasomes may be considered classical or non-classical. Classical inflammasomes require the participation of caspase-1, and include NLRP1, NLRP3, NLRC4, and AIM2. Non-classical inflammasomes are usually dependent on caspase-4 or caspase-5, and are less studied and need to be further explored (12). Classical pathways recruit pro-caspase-1 through ASC and transform it into active caspase-1. Activated caspase-1 promotes the conversion of pro-IL-1β and pro-IL-18 into the pro-inflammatory cytokines IL-1β and IL-18, resulting in apoptosis and pathogen clearance. This process plays an important role in innate immune defense (13) (**Figure 1**). IL-1β secreted extracellularly facilitates the pro-inflammatory effects of IL-6 and TNF-α, while IL-18 can mediate neutrophil maturation and local infiltration and induce the transformation of T-cells to the Th2 state. Th2 cells secrete IL-4 and IL-13, which can induce macrophages to differentiate into the M2 state and mediate anti-inflammatory effects (14).

### 2.1 NLR inflammasomes

#### 2.1.1 NLRP3 inflammasome

NLRP3, the most thoroughly studied inflammasome, consists of the sensor NLRP3, the adaptor protein ASC, and the effector protein caspase-1. The NLRP3 inflammasome is widely present in immune cells, including granulocytes, dendritic cells, macrophages, epithelial cells and osteoblasts (15). NLRP3 belongs to the family of NLR proteins and consists of three structural domains: a C-terminal leucine-rich repeat (LRR), a central nucleotide-binding domain (NBD), and an N-terminal PYD. The LRR is the key regulator of NLRP3 activity and senses endogenous damage and microbial presence. The NBD has ATPase activity and promotes self-oligomerization. The PYD is primarily involved in mediating downstream protein interactions. Normally, the NBD of NLRP3 binds to the LRR, inducing a state of self-inhibition. In the presence of PAMPs or DAMPs, NLRP3 exposes the NBD domain and oligomerizes. The PYD at the N-terminus of NLRP3 recruits PYD-bearing ASC splice proteins, and the CARD of ASC recruits pro-CARD-bearing caspase-1 to complete the assembly of the inflammasome (16).

The activation of the NLRP3 inflammasome requires two steps. In the first step (initiation), microbial or endogenous molecules such as TLR ligands activate NF-κB, which in turn induces the transcription and production of pro-IL-1β, pro-IL-18, and NLRP3. In the second step (activation), the NLRP3 inflammasome recognizes various PAMPs and DAMPs from diverse stimuli, such as fungi (10), bacterial (17), viruses (18, 19), fibrillar amyloid-β (Aβ) peptide (20), ATP (20), glucose (21), and so on. These stimuli promote the activation of the inflammasome and the maturation of pro-IL-1β and pro-IL-18. There are four main activation mechanisms of the NLRP3 inflammasome, including ionic flux, ROS production, mitochondrial dysfunction and lysosomal damage. In addition, atypical inflammasome activation can occur through a pathway stimulated by lipopolysaccharide. In mice, this process is predominantly controlled by caspase-11, while in humans, caspase-4 and caspase-5 are the main players. After the activation of caspase-4, caspase-5 or caspase-11, the intact gasdermin D (GSDMD) is cleaved to generate an N-terminal fragment, which binds to the cell membrane to form a pore, thus inducing pyroptosis (22). In a study aimed at identifying additional sensors for intracellular LPS by biochemical screening, the authors identified the orphan nuclear receptor Nur77 as an LPS-binding protein in macrophage lysates, which could bind mitochondrial DNA and LPS to activate the non-classical NLRP3 inflammasome (23). During microbial infections, the NLRP3 inflammasome can support the host immune system to resist infection, thus maintaining homeostasis. However, abnormal activation of the NLRP3 inflammasome causes excessive IL-1β and IL-18 production, which can trigger certain genetic or acquired inflammatory diseases.

#### 2.1.2 NLRC4 inflammasome

NLRC4, also known as IPAF (IL-1β converting enzyme protease activating factor), is similar to other NLRs and consists of three structural domains. The C-terminal LRR domain, consisting of 15 repetitive units with a total of 440 amino acids, with each unit connected to a helix structure containing 8-15 amino acids, recognizes ligands such as PAMP. The N-terminal CARD is usually composed of the first 94 amino acids of NLRC4, and folds into six inversely parallel α-helices wrapped around the hydrophobic core; the N-terminal CARD can connect the binding protein ASC and the effector molecule caspase-1, and mediate the downstream signaling. Finally, the central NBD, which includes a central NTPase domain, two helical domains (HD1, HD2) and the winged-helical domain (WHD). These protein domains occur in the order of NBD-HD1-WHD-HD2 from N-terminus to C-terminus, and can mediate the oligomerization of NLR molecules, thus changing their conformation (24).

The activation of the NLRC4 inflammasome is primarily regulated at the translational and post-translational levels by two main mechanisms: ligand binding and phosphorylation. In the event of bacterial infection, the LRR recognizes the ligands and undergoes a conformational change, facilitating the conversion of ADP to ATP. Consequently, the inhibition of NBD oligomerization by the LRR is removed, exposing the CARD and activating the NLRC4 inflammasome. Gram-negative pathogenic bacteria, such as *Salmonella typhimurium and Pseudomonas aeruginosa*, can activate the NLRC4 inflammasome by introducing flagellin or type three secretion system (T3SS) rod protein (PrgJ) into host cells through the transmembrane needle-like structure of the T3SS (25). Some NLRC4 proteins can also directly recruit and activate pro-caspase-1 without ASC, but require interaction with the NLR family apoptosis inhibitory protein (NAIP), which acts as a ligand to activate the NLRC4 inflammasome (26). After NLRC4 inflammasome activation, pro-caspase-1 can be recruited through the CARD of ASC or NLRC proteins to become the active caspase-1, which cleaves pro-IL-1β and pro-IL-18 into mature IL-1β and IL-18, resulting in further inflammation. In addition, caspase-1 can also activate GSDMD to cause pyroptosis. Pyroptosis not only induces host inflammation, but also captures pathogenic bacteria through cell membrane pores, thus promoting the host to initiate immune defense to eliminate the pathogenic bacteria (27).

#### 2.1.3 NLRP1 inflammasome

The NLRP1 inflammasome is a multi-protein complex composed of the receptor NLRP1, the adaptor protein ASC and the effector protein caspase-1. Humans have only one NLRP1 gene, which is similar to other NLRs but also has its own unique domains, consisting of an N-terminal PYD, a central NBD, an LRR, a function to find domain (FIIND) and a C-terminal CARD. In contrast, mice have three homologous NLRP genes: NLRP1a, NLRP1b, and NLRP1c, which are different from human NLRP1 in that they lack the N-terminal PYD and contain a truncated LRR, instead possessing an NR100 domain at the N-terminus, which activates the inflammasome and triggers subsequent pyroptosis when cleavage occurs (28). In the absence of no external stimulation, the NBD in NLRP1 binds to the LRR and inhibits self-oligomerization, resulting in an inactive state. However, when cells are exposed to activating substances that bind to the LRR in NLRP1, a conformational change in NLRP1 is induced, exposing the PYD and CARD. In general, PYD and CARD domains mediate other downstream proteins containing PYD and CARD, such as homotypic interactions with ASC and caspase-1, respectively. The NBD domain can interact with the LRR domain, and the FIIND domain enables NLRP1 to perceive the stability of its own proteins to detect and respond to pathogen-related activities. The unique CARD domain of NLRP1 can directly bind to caspase-1 through CARD-CARD interaction to complete the assembly of the NLRP1 inflammasome, so although ASC can promote NLRP1-mediated activation of caspase-1, it is not necessary for NLRP1 inflammasome activation (29). Oligomerized pro-caspase-1 is cleaved by auto-phosphorylation to form active caspase-1, which in turn promotes the cleavage of pro-IL-18 and pro-IL-1β to generate IL-18 and IL-1β, thereby inducing inflammation (30).

At present, several substances that activate the NLRP1 inflammasome have been identified, including bacterial cytosolic acyl dipeptide (MDP), anthrax lethal toxin (LT), and parasites (31). Recent studies have also shown that the expression of NLRP1 inflammasome in human lung epithelial cells is important for the detection of and response to SARS-CoV-2. However, SARS-CoV-2 infection can inhibit the classical caspase-1-GSDMD inflammasome pathway through its NSP5 protease (3CL protease) activity, while triggering epithelial cell pyroptosis *via* GSDME. Therefore, the NLRP1 inflammasome can sense SARS-CoV-2, but the NSP5 protease is capable of inhibiting innate immunity (32).

### 2.2 AIM2 inflammasome

The AIM2 protein is a member of the interferon-inducible HIN-200 protein family, and contains a PYD at its N-terminus that binds to the adaptor protein ASC, as well as two adjacent oligonucleotide/oligosaccharide binding domains (OB) at the C-terminus, which are associated with dsDNA recognition and binding (33). During the assembly of the AIM2 inflammasome, the PYD is responsible for the recruitment of the adaptor protein ASC. In the resting state, the PYD of AIM2 binds to the molecular complex within the HIN domain, thus maintaining the inhibitory state. However, when dsDNA binds to the HIN domain, a conformational change occurs, causing the PYD of AIM2 to bind directly and interact with the PYD of ASC. In turn, the CARD of ASC binds to the CARD of pro-caspase-1, promoting caspase-1 activation and the maturation of the downstream inflammatory cytokines IL-1β and IL-18 (34).

As an intracellular DNA receptor, AIM2 can recognize both viral DNA that invades host cells and bacterial DNA that exerts an escape mechanism to enter the cytoplasm by punching holes in the phagosomal membrane; both sources of dsDNA act as ligands to activate AIM2. By recognizing dsDNA, the AIM2 inflammasome plays an indispensable role in host innate immunity against pathogenic microorganisms. AIM2 not only recognizes bacterial and viral dsDNA in the cytoplasm, but also detects damaged and mislocalized DNA molecules (35). However, AIM2 activation can be inhibited by repeated TTAGGG sequences, which are often seen in mammalian telomeric DNA and function to maintain the inhibitory state of AIM2 by competing with dsDNA. Synthetic DNA containing this sequence also inhibits the activation of the AIM2 inflammasome (36). In addition, POP1 and POP3 proteins of the PYD can also inhibit ASC-dependent inflammasome assembly by preventing inflammasome nucleation, thus inhibiting AIM2 inflammasome activation and interfering with caspase-l activation and the release of IL-1β and IL-18 (37).

## 3 The dual roles of inflammasomes in tumorigenesis and development

The inflammasome is a molecular platform that accumulates in response to various intracellular stimuli and may influence tumorigenesis by regulating both innate and adaptive immune responses. Aberrant inflammasome activation is associated with inflammation, metabolic diseases, neurodegeneration and malignant tumors. Inflammasomes play a dual role in the occurrence and development of different tumors (**Table 1**). On the one hand, the activation of inflammasomes can trigger an effective immune response to limit the invasion of pathogens, while also inducing cell death under stress and inflammatory pathological conditions by regulating caspase-1-dependent cell pyroptosis (56). On the other hand, dysregulated activation of inflammasomes could lead to a hyperinflammatory state, creating an environment that encourages tumor growth and promotes metastasis (57). The specific role of inflammasomes is influenced by their expression level, downstream effector molecules, tumor types and stages of tumor growth.

**Table 1:** The dual role of inflammasomes in different types of cancers.

	The type of cancer	Inflammasome	The effects of the inflammasome	Reference
Inflammaso-mes in tumor suppression	colitis-associated colon cancer	NLRP3	Inhibition of tumorigenesis through activation of ASC, IL-18 and caspase1	(38, 39)
	lung adenocarcinoma	NLRP1	Inhibition of tumorigenesis by increasing the expression level of NLRP1	(40)
	colorectal cancer	NLRP1	Inhibition of tumorigenesis through regulation of IL-18 and IL-1β	(41)
		NLRC4	Inhibition of tumorigenesis by suppressing cell proliferation	(42)
		AIM2	Inhibition of tumorigenesis by impairing colon cancer stem cell proliferation and promoting cell death	(43)
	malignant melanoma	NLRC4	Inhibition of tumorigenesis by promoting the production of IFN-γ in CD4^+^ and CD8^+^ T cells	(44)
	breast cancer	AIM2	Inhibition of tumorigenesis by leading to cancer cell apoptosis	(45)
	liver cancer	AIM2	Inhibition of tumorigenesis by suppressing the mTOR-S6K1 signaling pathway	(46)
Inflammaso-mes in tumor promotion	metastatic melanoma	NLRP3	Promotes tumorigenesis by suppressing NK cell and T cell activity and recruiting MDSCs and Tregs	(47)
		NLRP1	Promotes tumorigenesis by enhancing NLRP1 inflammasome activation and inhibiting apoptosis	(48)
	lung adenocarcinoma	NLRP3	Promotes tumorigenesis by recruiting tumor-associated macrophages and releasing IL-1β and IL-1α	(49)
		AIM2	Promotes tumorigenesis by regulating the expression of the cell cycle proteins through the AIM2/IL-1β/STAT3 signaling pathway	(50)
	breast cancer	NLRP3	Promotes tumorigenesis through activation of NLRP3 and release of IL-1β	(51)
		NLRC4	Promotes tumorigenesis through adipocyte-mediated VEGFA expression	(52)
		NLRP1	Promotes tumorigenesis by inducing epithelial-mesenchymal transformation	(53)
	multiple myeloma	NLRP1	Promotes tumorigenesis by releasing IL-18 and increasing the production of MDSCs	(54)
	cutaneous squamous cell carcinoma	AIM2	Promotes tumorigenesis by increasing the expression of cell cycle regulatory genes	(55)

### 3.1 The suppression of tumorigenesis and tumor development by inflammasomes

**Figure 2:**
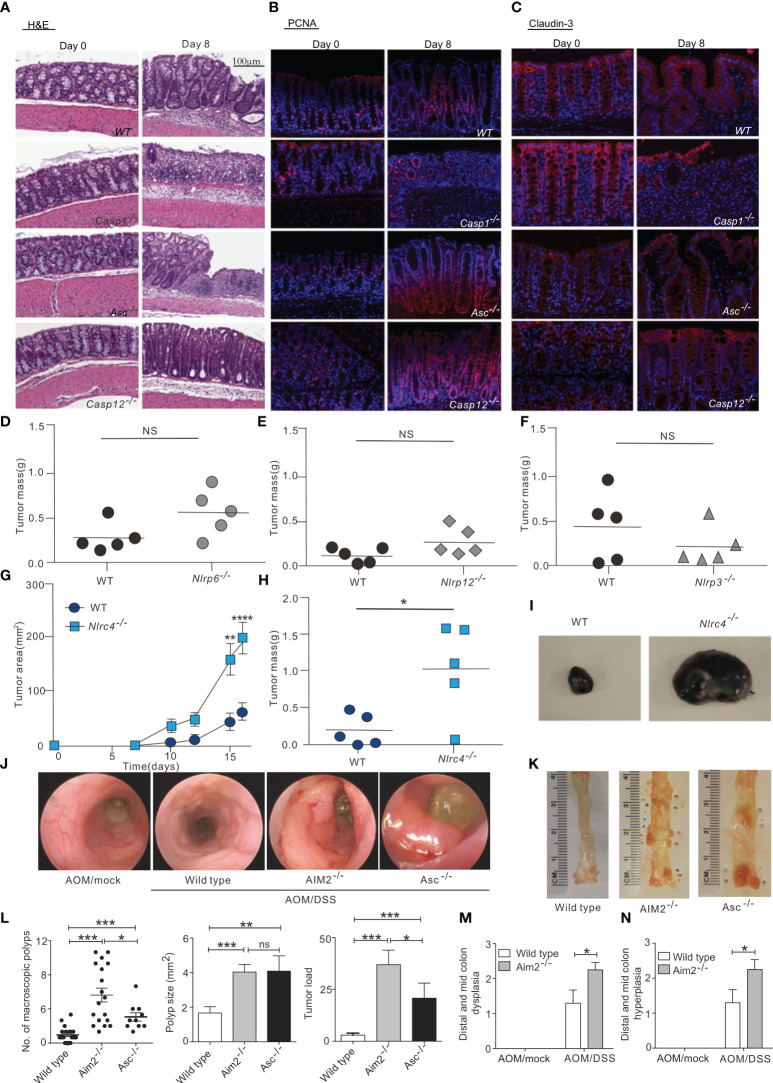
Inflammasome in tumor suppression. **(A)** Hematoxylin and eosin (H&E) staining of colon sections derived from WT, *Casp1^-/-^*, *Asc^-/-^* and *Casp12 ^-/-^* mice on days 0 and 8 after the start of DSS treatment (magnification 100×). **(B, C)**, Immunofluorescence was performed on colon sections derived from WT, *Casp1^-/-^*, *Asc^-/-^* and *Casp12 ^-/-^* mice on days 0 and 8 for examination of proliferating cells (PCNA) **(B)** and tight junction (Claudin-3) **(C)**; (magnification 100×). , WT and *Nlrp6^–/–^* **(D)**, *Nlrp12^–/–^* **(E)**, *Nlrp3^–/–^* **(F)**, and *Nlrc4^–/–^* **(G–I)** mice were injected s.c. with 1 × 10^5^ B16F10 cells. **(D-F, H)** Tumor mass was determined at 16 to 20 days after inoculation. **(G)** WT and *Nlrc4^–/–^* tumor areas (length × width) were measured every 2 to 3 days. **(I)** Representative images of excised WT and *Nlrc4^–/–^* B16F10 tumors. **(G, H)** Data are representative of 3 independent experiments with n = 5 mice per group. **(J, K)** Mini-endoscopy **(J)** and representative images **(K)** of colons from AOM/DSS-treated wild-type (n = 5), *Asc^−/−^* (n = 3) and *Aim2^−/−^* mice (n = 5). **(L)** Macroscopic polyp counts, average polyp size per mouse and tumor load (n = 21 for WT, n = 18 for *Aim2^−/−^* and n = 10 for *Asc^−/−^*). **(M, N)** Semiquantitative scoring of colon hyperplasia **(M)** and dysplasia **(N)** in AOM/DSS-treated wild-type and *Aim2^−/−^* mice (n = 6 for WT AOM/mock and *Aim2^−/−^* AOM/DSS, and n = 5 for WT AOM/DSS and *Aim2^−/−^* AOM/mock). The figures A–C are reprinted with permission from Ref (39). Copyright Cell Press. The figures D–I are reprinted with permission from Ref (44). Copyright American Society for Clinical. The figures **(J–N)** are reprinted with permission from Ref (43). Copyright Nature Publishing Company. *P<0.05, **P<0.01, ***P<0.001, ns: no significance.

NLRP3 is the most widely studied inflammasome that not only regulates the tumor itself, but also influences the composition of the tumor microenvironment. The biological importance of the NLRP3 inflammasome in many diseases, including colorectal cancer, melanoma and metastases, is dependent on its ability to respond to multiple signals. In a mouse model of azoxymethane (AOM)-dextran sulfate sodium (DSS)-induced colon cancer, NLRP3 knockout mice were more likely to develop colon polyps and were highly sensitive to AOM-DSS-induced colitis-associated colon cancer. Moreover, tumor growth was observed to be accelerated in NLRP3 knockout mice, concomitant with decreased IL-18 levels in the tissue. IL-18 helps repair the epithelial barrier to counteract damage, and after AOM-DSS induction, IL-18^-/-^ mice were more likely to develop tumors than control mice, indicating that IL-18 has potential anti-tumor effects (38). In addition, ASC and caspase-1 knockout mice are also susceptible to DSS-induced colitis and colitis-associated colon cancer, suggesting that the activation of the NLRP3 inflammasome may have an inhibitory effect on colorectal carcinogenesis (39) (**Figures 2A–C**). Furthermore, IL-18 secretion mediated by the NLRP3 inflammasome can indirectly inhibit the tumor progression of colitis-associated colorectal cancer by inducing regulatory T (Treg) cells to produce IFN-γ and enhancing the cytotoxicity of T cells and NK cells (58). Hence, the presence of the NLRP3 inflammasome is also necessary in the anti-tumor adaptive immune response. Moreover, chemotherapy treatment of tumor cells (e.g., with oxaliplatin and anthracyclines) can cause tumor cells to release ATP, which is an activation signal for the NLRP3 inflammasome and the IL-1β/IL-1 receptor (IL1R) signaling axis in dendritic cells (DCs) (59). Subsequent P2X7 receptor binding drives the anti-tumor immune response of CD8^+^ T cells against transplanted tumors (41). In addition, the activation of inflammasomes may enhance the therapeutic effect of anti-PD-1 antibody immunotherapy by activating CD8^+^ T cells. Activated CD8^+^ T cells may further promote the activation of NLRP3 inflammasomes of antigen-presenting cells through perforin-dependent mechanisms and achieve anti-tumor effects through a positive feedback mechanism (60).

Similarly, NLRP1 inflammasome activity can also influence cancer growth. In inflammatory bowel disease and colorectal cancer, the NLRP1 inflammasome exerts effects through IL-1β and IL-18. NLRP1 expression levels were downregulated in biopsied tissues of colon cancer patients compared with healthy controls, suggesting that NLRP1 can act as a key regulator of colon homeostasis (41). Additionally, a biological analysis of lung adenocarcinoma (LUAD) data from the TCGA and GEO databases found that the levels of NLRP1 mRNA were significantly lower in LUAD tissues than in non-cancerous lung tissues, and that LUAD progression was negatively associated with NLRP1 expression, in terms of clinical features such as tumor pathological stage, lymph node metastasis, and primary tumor status. In addition, NLRP1 has been positively correlated with the degree of infiltration of tumor-infiltrating immune cells (40). Similarly, the NLRC4 inflammasome can also inhibit the occurrence of colorectal cancer by suppressing cell proliferation and promoting cell death (42). The NLRC4 inflammasome has also been found to enhance inflammatory signaling pathways in macrophages *via* non-inflammasome pathways and promote the production of IFN-γ in CD4^+^ and CD8^+^ T cells, thereby inhibiting the growth of melanoma in mice (44) (**Figures 2D–I**).

In addition to the NLR family, the AIM2 inflammasome, which recognizes DNA, was also shown to inhibit AOM-DSS-induced colorectal cancer and spontaneous colorectal carcinogenesis by impairing colon cancer stem cell proliferation and promoting cell death (43) (**Figures 2J–N**). AIM2 is an interferon-inducible protein and contains HIN-200, which can inhibit the cell cycle and hinder tumor growth. Studies have shown that AIM2 can inhibit cell proliferation and also promote apoptosis when expressed in large amounts by inducing the release of cytochrome c from the mitochondria to the cytoplasm. In breast cancer, overexpression of AIM2 can decrease the expression of the anti-apoptotic protein Bcl-xl, increase the expression of the pro-apoptotic Bax, and induce the cleavage of the DNA repair protein PARP, thus leading to breast cancer cell apoptosis and the inhibition of breast cancer progression (45). Compared with the terminally differentiated, normal, cancer-free tissues, the expression of AIM2 in hepatocellular carcinoma tissues was significantly reduced, and AIM2 expression was negatively correlated with tumor burden. AIM2 may inhibit hepatocarcinogenesis in immunocompromised nude mice by suppressing the mTOR-S6K1 signaling pathway. Studies involving AIM2 silencing and overexpression in hepatocellular carcinoma cells further revealed that AIM2 exerts anti-tumor effects through inflammasome activity leading to pyroptosis (46).

Hence, inflammasomes can inhibit tumor occurrence and development in certain types of cancer. These tumor suppressive activities depend on the ability of the inflammasome to produce and regulate cytokines, especially those that affect T-cell activity and cell proliferation and maturation.

### 3.2 The promotion of tumorigenesis and development by inflammasomes

However, despite their tumor-suppressive effects in some contexts, it has also been shown that inflammasomes can contribute to tumor development by influencing host tumor immunity, promoting tumor cell proliferation and differentiation, and regulating the tumor microenvironment.

**Figure 3:**
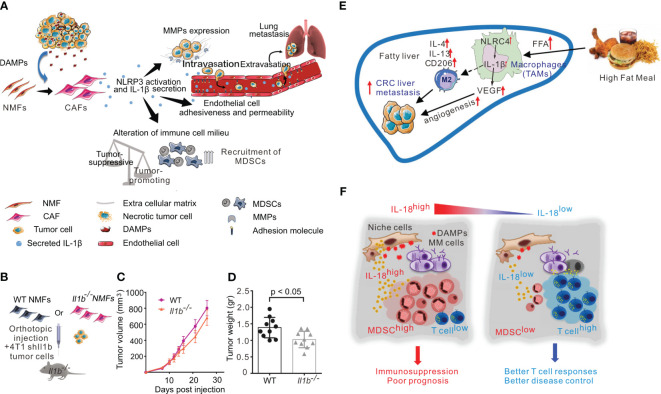
Inflammasome in tumor promotion. **(A)** Activation of the NLRP3 inflammasome in cancer-associated fibroblasts links tissue damage with tumor-promoting inflammation in breast cancer progression and metastasis. **(B–D)**: CAF-derived IL-1β facilitates lung metastasis. **(B)**Scheme of experiments analyzed in B-D. shIl1b 4T1 mammary carcinoma cells were orthotopically coinjected with WT NMFs (*Il1b^+/+^*) or with *Il1b^−/−^* NMFs into the right inguinal mammary glands of *Il1b^−/−^* female mice. **(C)** Growth curves of 4T1 tumors injected with WT NMFs (*Il1b^+/+^*) or with *Il1b^−/−^* NMFs. n = 10 mice per group. **(D)**Tumor weight at termination of experiment. n = 10 mice per group. **(E)** In NAFLD, NLRC4 contributes to M2 polarization, IL-1β, and VEGF production in TAMs, which promote metastatic liver tumor growth. **(F)** The pro-inflammatory cytokine IL-18 was critically involved in MM-associated inflammation and immunosuppression, and dysregulated IL-18 was a potential therapeutic target in the MM niche. The figures A-D are reprinted with permission from Ref (51). Copyright Nature Publishing Group. The figure E is reprinted with permission from Ref (62). Copyright WILEY. The figure F is reprinted with permission from Ref (55). Copyright Cell Press.

The tumor microenvironment includes proliferating tumor parenchymal cells, vascular endothelial cells, inflammatory infiltrating cells, and associated stromal cells, as well as their secreted proteins and cytokines. Among these, pro-inflammatory immune cells are a key component and play an important role in tumorigenesis, proliferation, progression, invasion, and metastasis (61). Myeloid-derived suppressor cells (MDSCs), a key component of the tumor microenvironment (TME), produce anti-inflammatory cytokines and encourage Treg proliferation, both of which have strong immunosuppressive activity. In mouse models of sarcoma and metastatic melanoma, activated NLRP3 inflammasome promotes tumorigenesis by suppressing NK cell and T cell activity and recruiting MDSCs and Tregs. Notably, in NLRP3-/- mice, a 5-fold reduction in the number and activity of MDSCs was found, suggesting that NLRP3 affects tumorigenesis by modulating host immunity (47). Tumor-associated macrophages (TAMs) are among the immune cells that infiltrate into tumors and play an important role in tumor lymphangiogenesis and proliferation, TAMs are highly aggregated in the tumor microenvironment of non-small cell lung cancer (NSCLC). The NLRP3 inflammasome in the cytoplasm of lung TAMs initiates TLR4/caspase-1 and TLR4/caspase-11 signaling pathways through TLR4, which are involved in the release of IL-1β and IL-1α, and promote lung tumor formation (49). Ershaid et al. found that cancer-associated fibroblasts can sense DAMPs and activate the NLRP3 inflammasome, which in turn triggers IL-1β secretion and further promotes the growth and metastasis of mouse and human breast cancer cells. However, when NLRP3 or IL-1β is silenced, the tumor-promoting effect is impaired. These results suggest that NLRP3 can induce the growth, metastasis and invasion of breast cancer and increase its incidence and development (51) (**Figures 3A–D**).

Obesity is known to increase the risk of cancer, and inflammasome activity may partially account for this observation. Kolb et al. found that NLRC4 inflammasome/IL-1β signaling promotes the progression of breast cancer. In obesity, the TME causes NLRC4 inflammasome activation in TAMs, leading the production of the pro-inflammatory cytokine IL-1β, which then promotes the angiogenesis of trophoblast tumor cells through adipocyte-mediated vascular endothelial growth factor A (VEGFA) expression (52). In a mouse model of non-alcoholic steatohepatitis (NASH), NLRC4 knockout promoted the growth and recurrence of liver metastases of colorectal cancer, but significantly restricted hepatic tumorigenesis. NLRC4^-/-^ mice with NAFLD also had decreased TAMs and decreased IL-1β and VEGF expression, suggesting that NLRC4 plays a key role in the growth and recurrence of liver metastases in the context of both NAFLD and colorectal cancer (62) (**Figure 3E**).

NRLP1 has been implicated in melanoma, with one study examining 216 melanoma tissue samples and 13 human melanoma cell lines to report that NLRP1 expression was elevated in melanoma. Meanwhile, knockdown of NLRP1 in human metastatic melanoma cells decreased caspase-1 activity, IL-1β secretion and NF-κB activity. Futhermore, the activity of caspase-2 and caspase-9 were enhanced and apoptosis was promoted. Therefore, there is evidence to suggest that NLRP1 promotes melanoma growth by enhancing inflammasome activation and inhibiting apoptosis in melanoma cells (48). The NLRP1 inflammasome has also been studied in the TME of multiple myeloma, where NLRP1 increases the production of MDSCs by sensing disturbances in hematopoietic stress or cellular homeostasis and responding by secreting IL-18, which leads to accelerated tumor progression (54). High expression levels of NLRP1 have also been found in human breast cancer mcf-7 cells and breast cancer tissues, and were correlated with lymph node metastasis, tumor lymph node metastasis stage and Ki-67 levels. In nude mouse transplantation models of breast cancer, overexpression of NLRP1 was found to promote breast cancer migration, invasion and growth by inducing epithelial-mesenchymal transformation (53).

In addition to the NLR family inflammasomes, AIM2 has also been implicated in cancer growth. Dysregulation of pro-inflammatory cytokines in the lung is one of the most important causes of inflammatory diseases and NSCLC. In NSCLC cells, AIM2 overexpression has been shown to enhance cell viability and migration. AIM2 also played an oncogenic role to regulate the expression of the cell cycle proteins cyclinB1 and cDC2 through the AIM2/IL-1β/STAT3 signaling pathway, thereby regulating NSCLC cell cycle progression (50). In the context of skin cancer, primary and metastatic cutaneous squamous cell carcinoma (cSCC) were found to exhibit higher levels of AIM2 expression than normal skin. AIM2 inflammasome activity can cause cSCC cells to release indirect effector cytokines and increase the expression of cell cycle regulatory genes, such as CDK1, CDC7, and CCNA1. Consequently, cell death is inhibited and the proliferation rate is increased, leading to rapid tumor development. Conversely, AIM2 knockdown decreased CDK1 expression, tumor cell invasion, and metastatic potential, and inhibited the growth and angiogenesis of metastatic tumor cells. Therefore, AIM2 is likely to promote the occurrence and metastasis of cSCC and can be used as a therapeutic target for cSCC (55) (**Figure 3F**).

To sum up, the role of inflammasomes in tumor growth metastasis is bidirectional. The precise function of any one inflammasome in a specific context depends on a variety of factors, including the type of cancer and the route of inoculation.

## 4 Inflammasomes and cancer therapy

In recent years, there have been major advances in cancer immunotherapy, which now involves multiple modes of action. Therapeutic strategies include targeting specific cells, regulating the TME, systemic administration of immunosuppressive cells, and influencing immune checkpoint inhibitors, among others (63). T cells and NK cells remain the primary targets for tumor treatment, as T cells can exhibit antigen-specific tumor cell-targeting and NK cells can kill both tumor cells and virally infected cells. Additionally, antigen-presenting cells (APCs) remain central to enhancing the anti-tumor response (64). Inflammasomes play an important role in the regulation of tumor immunity, as the inflammatory response can frequently stimulate the maturation and antigen presentation of APCs such as DCs. Hence, inflammasomes can be targeted or modulated to achieve tumor immunotherapy.

### 4.1 Inflammasomes in immune cells during cancer treatment

Chemotherapeutic drugs (such as anthracyclines and platinum drugs) selectively kill immunosuppressive cells such as Tregs, MDSCs and TAMs, similar to inflammasome activity, which also regulates the death of immunosuppressive cells (65). The NLRP3 inflammasome has been observed to be activated in DCs during chemotherapy and enhanced the anthracycline-induced anti-tumor immune response by secreting caspase-1 and IL-1β. However, the release of various DAMPs, including ATP, HMGB1 and IL-1α, from dead tumor cells activates the NLRP3 inflammasome in DCs, which can lead to resistance to chemotherapy and promote tumor growth (66). In addition, gemcitabine and 5-fluorouracil (5-FU) can trigger the release of cathepsin B in MDSCs and activate the NLRP3 inflammasome. Although the level of IL-1β that is subsequently produced cannot activate CD8^+^ T cells, they can induce CD4^+^ T cells to produce IL-17, thus promoting tumor growth and angiogenesis (67).

Recent studies have shown that the expression of NLRP3, AIM2 and caspase-1 was significantly increased in tumor specimens from melanoma patients who had been effectively treated with PD-1. Although this study cannot show a causal relationship between inflammasome-secreted IL-1 β or IL-18 and the efficacy of PD-1 treatment, it nevertheless provides observational evidence that NLRP3 expression was associated with increased numbers of CD8^+^ T cells and memory CD4^+^ T cells (68). After treatment with PD-1 inhibitors, *in vivo* and *in vitro* studies have shown that the activation of CD8^+^ T cells can cause a signaling cascade mediated by endogenous PD-L1/NLRP3 inflammasomes, resulting in the recruitment of polymorphonuclear-MDSCs into tumor tissues, thereby suppressing the anti-tumor immune response (69). TIM-3 is another emerging immune checkpoint molecule similar to PD-1/PD-L1 and CTLA-4. Specific knockdown of TIM-3 in DCs resulted in significant inhibition of tumor growth and improved the antigen-presenting ability of DCs. In addition, single-cell sequencing data showed that inflammasome-related genes such as AIM2, NLRP3, IL-1β and caspase1 were significantly enriched in TIM-3 knockout DCs. Changes in cellular function in the TME and altered molecular pathways in DCs suggested that TIM-3 deficiency activates inflammasomes in DCs, which maintains the proliferative activity of CD8^+^ T cells and increases their tumor-killing capacity (70).

The NLRP3 inflammasome can also impair the efficacy of anti-tumor vaccines. In a mouse model of melanoma, NLRP3^-/-^ mice that were vaccinated with DC-based vaccines had significantly increased survival compared to wild-type controls, possibly due to the presence of fewer MDSCs in NLRP3^-/-^ mice, which may have contributed to impaired tumorigenesis (47). Knockout of the NLRP3 inflammasome also induced NK cell infiltration, increased production of chemokines CCL5 and CXCL9, and enhanced anti-metastatic activity of NK cells. Therefore, targeted inhibition of inflammasome activation can reduce the number of immunosuppressive cells and thus inhibit tumor growth and metastasis.

### 4.2 Targeting inflammasomes to modulate cancer therapy

Tumor progression is not solely dependent on the behavior of cancer cells, but regulated by a complex plethora of cellular and non-cellular signals from the inflammatory microenvironment. As discussed above, inflammasomes can promote carcinogenesis, but they are also an ideal target for the prevention and treatment of tumors. There are several approaches to target inflammasome activity, including blocking upstream signaling pathways, inhibiting inflammasome components and antagonizing the end products of inflammasome activation (71) (**Table 2**). However, the use of synthetic compounds or small molecule inhibitors that target inflammasomes and their components is limited by poor specificity and low efficiency. In clinical studies, inhibition of cytokine signaling has been the most successful approach. For example, monoclonal antibodies that block IL-1R (e.g., anakinra, rilonacept, canakinumab, and gevokizumab) have been used to impair IL-1α- and IL-1β-mediated signaling *via* IL-1R, for the prevention or treatment of multiple myeloma through inhibition of IL-1β-induced production of IL-6 (72).

**Table 2:** Inflammasomes inhibitors.

Therapeutic agents	Targets	Potential Mechanism	Diseases
Anakinra	IL-1 receptor	Recombinant IL-1Ra	RA, Destructive joint process Autoimmune disease (FMF, CAPS, HIDS, TRAPs), Systemic/common inflammatory diseases (Gout, T2D, Systolic heart failure)
Rilonacept	IL-1β	Extracellular portion of the IL-1R and the Fc domain of human IgG1	CAPS, Diabetes, Gout, Recurrent Pericarditis
Canakinumab	IL-1β	Anti-IL-1β antibody	MWS, FCAS
Ritonavir	Caspase-1	Protease inhibitor	Pancreatitis
Avastin	P2X7	P2X7 inhibitor	Solid tumor
Glyburide	NLRP3 (Indirect)	Broad-spectrum inhibitor of the ATP-binding cassette transporter and P2X7R	Type2 diabetes
Gevokizumab (Xoma 052)	IL-1β	Anti-IL-1β antibody	Diabetes, Osteoarthritis
GSK 1070806	IL-18	Anti-IL-18 antibody	Diabetes, IBD
Ac-YVAD-CHO	Caspase-1	Caspase-1 inhibitory peptide	Endotoxemia, Pancreatitis
VX-765	Caspase-1	Selective inhibitor of caspase-1	CAPS, MWS, RA
Pralnacasan (VX-740)	Caspase-1	Caspase-1 inhibitor	RA, OA
Thalidomide		Caspase-1 inhibitor	Myeloma
CRID3	ASC	Blocking putative association between GSTO1 and ASC	IBD
MCC950	NLRP3	Inhibitor of ASC oligomerization	experimental autoimmune encephalomyelitis (EAE)
AZD9056	P2X7	Inhibitor of NLRP3 ATPase	RA
GSK1482160	P2X7	P2X7 antagonist	RA
Bay 11-7082	NF-κB/NLRP3 ATPase	Inhibitor of NLRP3 ATPase	psoriasis
Parthenolide	Caspase-1/NF-κB NLRP3 ATPase	Inhibitor of NLRP1/3/4 or caspase-1, inhibitor of NLRP3 ATPase	Dermatitis
16673-34-0	NLRP3 (Indirect)	Glyburide derivatives	Acute myocardial infarction
β-Hydroxybutyrate (BHB)	NLRP3 (Indirect)	Inhibits K+ efflux resulting in reduced oligomerization ASC and IL-1β/18 release	MWS, FCAS, AD
JC124	NLRP3	blocks ASC aggregation, caspase-1 activation, and IL-1β secretion.	acute myocardial infarction
FC11A-2	NLRP3	hindered the proximity-induced autocleavage of procaspase-1	experimental colitis
CY-09	NLRP3 ATPase	directly binds to the ATP-binding motif of NLRP3 NACHT domain and inhibits NLRP3 ATPase activity	CAPS, T2D
OLT1177	NLRP3 ATPase	reduced ATPase activity of NLRP3	CAPS
Tranilast	NLRP3 oligomerization	directly binds to the NACHT domain of NLRP3 and blocks NLRP3 oligomerization	CAPS, T2D
Oridonin	Cysteine 279 of NLRP3	block the interaction between NLRP3 and NEK7	peritonitis, gouty arthritis, T2D

Avastin (bevacizumab) is a specific inhibitor of the P2X7 receptor, a trimeric ATP-gated channel essential for the NLRP3/caspase-1 cascade. By blocking NLRP3 signaling, Avastin effectively inhibits tumor growth. Avastin has been approved by the FDA for the treatment of breast, colon, and lung cancers. Avastin inhibits proliferation and anti-apoptotic gene expression in tumor cells, while also preventing the activation of the transcription factor NFATc1 and suppressing the production of vascular endothelial growth factor (VEGF) (73). Glibenclamide is another chemotherapy drug that influences NLRP3. Glibenclamide inhibits K^+^ influx and thereby suppresses the maturation of caspase-1 and pro-IL-1β in macrophages. In human trophoblast cells, glibenclamide acts downstream of the P2X7 receptor and upstream of NLRP3, effectively blocking the activation of the NLRP3 inflammasome (74).

Other inflammasome-targeting therapies in development include MCC950, a small molecule NLRP3 inflammasome inhibitor. MCC950 has been shown to prevent both typical and atypical NLRP3 inflammasome activation and the secretion of IL-1β in a mouse model of experimental autoimmune encephalitis (EAE). However, MCC950 not only alleviates the symptoms of EAE, but also inhibits tumor development, indicating that it may have potential use for the treatment of NLRP3 inflammasome-related cancer (75). For instance, Chen et al. showed that the application of MCC950 in a mouse model of head and neck squamous cell carcinoma (HNSCC) significantly reduced IL-1β production in tumors. MCC950 also reduced the number of MDSCs, Tregs, and TAMs, depleted PD-1^+^ and Tim3^+^ T cells, and increased the number of CD4^+^ and CD8^+^ T cells. These results suggest that the NLRP3/IL-1β pathway promotes the development of HNSCC and that modulating of the tumor microenvironment by targeting the NLRP3/IL-1β pathway is expected to be a new therapeutic approach for HNSCC (76). In addition, in mouse macrophages and DCs, microRNA-223 inhibits IL-1β secretion and NLRP3 expression by directly binding to the 3’ untranslated region (3’UTR) of the NLRP3 mRNA, thus suppressing NLRP3 inflammasome activation (77). Some other microRNAs, including microRNA-155, microRNA-377, and microRNA-133a-1, are also involved in NLRP3 inflammasome activation and are expected to exert inhibitory effects. Another small molecule inhibitor, andrographolide, slows down the development of colitis-associated colon cancer (CAC) in mice by mediating mitochondrial phagocytosis and inhibiting NLRP3 inflammasome activation in macrophages (78). Therefore, novel treatments that target inflammasome activation may have a role in tumor therapy.

**Figure 4:**
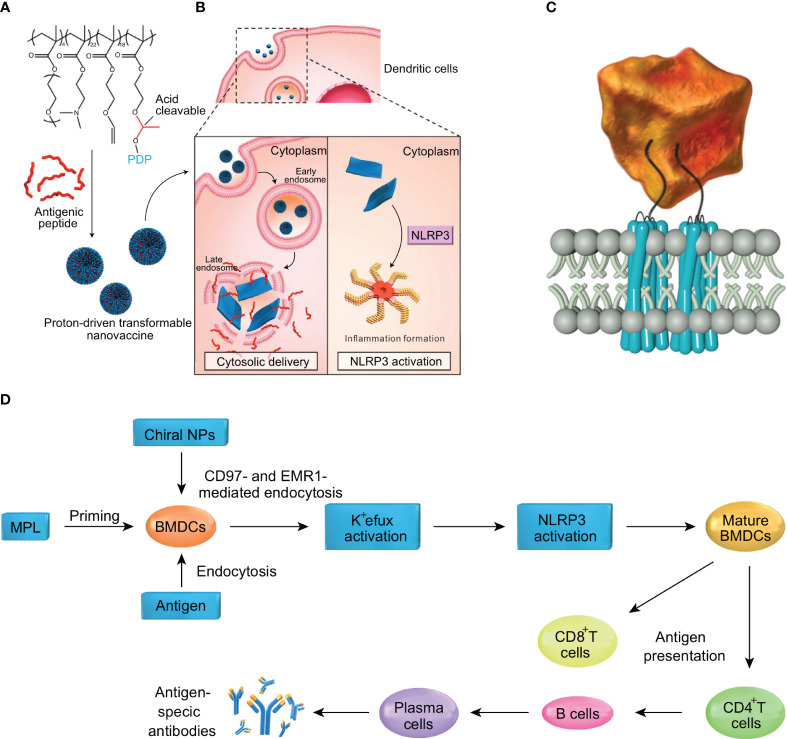
Nanoparticles by activating inflammasomes enhance antitumor immunity. **(A)** The nanotransformer-based vaccine (NTV) is composed of a polymer–peptide conjugate-based nanotransformer (NT) loaded with antigenic peptide (AP). The NTV has a spherical morphology with a diameter of about 100 nm at pH 7.4. **(B)** After the NTV is internalized by DCs, the acidic endosomal environment (pH 5.6) will trigger fast cleavage of the pyrene-conjugated D-peptide (PDP), which will then re-assemble into nanosheets with sizes in the range 5–8 μm. The morphological change leads to disruption of the endosomal membrane and delivery of AP into the cytosol. Moreover, the cytoplasmic nanosheets activate the NLRP3 inflammasome pathway, which promotes DC maturation and antigen processing. These two features contribute to the enhanced cross-presentation of AP to CD8^+^ T-cells and efficient antitumor immunity. **(C)** Diagram showing the interaction of chiral NPs with extracellular chiral chains of EGF-like domains on cellular AGPCR receptors. **(D)** The mechanism of induction of immune responses by chiral NPs. The figures A and B are reprinted with permission from Ref (83). Copyright John Wiley & Sons Inc. The figures C and D are reprinted with permission from Ref (82). Copyright Nature Publishing Group.

To optimize drug delivery, advances in nanomaterials have demonstrated promising results in the field of biomedicine. Nano-drug delivery systems have been widely used in oncology and immunotherapy, as encapsulating drugs in nanoparticles can improve the stability of hydrophobic drugs and reduce toxicity (79). However, the size, shape, surface charge and other physicochemical properties of nanoparticles can affect the activation of inflammasomes. Lunov et al. found that amine-modified polystyrene nanoparticles (PS-NH2) can trigger the assembly of NLRP3 inflammasomes and promote the downstream release of pro-inflammatory factor IL-1β from human macrophages (80). Similarly, silica nanoparticles (SiNPs) have also been shown to activate NLRP3 inflammasomes *in vitro*, resulting in the production of pro-inflammatory cytokines IL-1β and IL-18 (81). Moreover, studies have shown that chiral adjuvants can activate the NLRP3 inflammasome pathway by specifically binding to G protein-coupled receptors on the surfaces of antigen-presenting cells. Consequently, cytokines such as IL-2, IL-4, IL-12, and IFN-γ are released, simultaneously inducing cellular and humoral immune responses to achieve lasting vaccine protection (82) (**Figures 4C, D**). Other researchers have proposed an anti-tumor vaccine based on a proton-driven deformable nano-delivery system, which not only efficiently delivers antigen peptides into the cytoplasm of immune cells, but also enhances the innate immune system by acting as an adjuvant to activate the NLRP3. Deformable nano-tumor vaccine delivery systems can effectively inhibit the growth of mouse melanoma (B16) and human papillomavirus (HPV) tumors. In addition, the combination of this deformable nano-vaccine and anti-PD-L1 antibody therapy could significantly inhibit tumor growth in tumor-bearing mice, with about half of the mice exhibiting complete tumor regression *in vivo* (83) (**Figures 4A, B**). However, despite the beneficial effects of NLRP3 inflammasome activation in anti-tumor vaccine therapy, the activation of NLRP3 inflammasomes by nanoparticles may also have adverse effects for cancer treatment due to the release of large quantities of pro-inflammatory cytokines (e.g., TGF-β). Cytokine treatment of human cervical cancer cells (Hela), alveolar epithelial adenocarcinoma cells (A549) and ovarian epithelial adenocarcinoma cells (SKOV3) has been shown to result in significantly increased levels of epithelial-mesenchymal transformation. In addition, spontaneous metastasis of subcutaneously implanted mouse lung squamous carcinoma cells (KLN 205) was observed after intravenous injection of nanoparticles into tumor-bearing mice, whereas no spontaneous metastasis occurred in the control group (84). To sum up, nanomaterials play an important role in the activation of inflammasomes. While multi-functional small molecular nanomedicines show strong potential for targeted therapy of malignant tumors, their application in clinical practice should be undertaken with caution.

## 5 Conclusions and perspectives

Inflammasomes can be divided into classical and non-classical categories. Classical inflammasomes require the participation of caspase-1, and include NLRP1, NLRP3, NLRC4, and AIM2, which are widely studied at present, among which the NLRP3 inflammasome is the most thoroughly studied. Non-classical inflammasomes are mainly dependent on caspase-4 or caspase-5, and there are few studies in this area, so further exploration is required. NLRP11 has been found to be an important component of the NLRP3 inflammasomes in human macrophages, which may have been overlooked in the past as mouse cells do not contain NLRP11 and the NBD structural domain between NLRP11 and NLRP3 does not bind specifically in other human cells such as HEK293 cells. NLRP11 interacts with NLRP3 and ASC to prevent the assembly of NLRP3 inflammasomes, the polymerization of NLRP3 and ASC, the activation of caspase-1, pyroptosis and the release of cytokines. NLRP11 knockout can specifically influence the activation of NLRP3 inflammasomes without affecting the activation of other inflammasomes (85), further indicating the complexity of the regulation of different inflammasome complexes. Hence, although inflammasomes are highly conserved in evolution, attention should be paid to their species-specificity.

The numerous pathways regulated by inflammasome activation are complex and diverse, pertaining to several aspects of cell function. First, multiple modalities of regulation are present simultaneously, such as post-transcriptional regulation and post-translational modification, including phosphorylation and ubiquitination. Second, the same mode of regulation may mediate completely opposite results. Furthermore, different regulatory proteins or small molecules may act on the same site of the inflammasome and exert the same or opposite effects. Finally, the same regulatory mechanism may act on different inflammasomes to produce different outcomes.

Future research will undoubtedly shed further light on the molecular and cellular mechanisms by which inflammasomes and immune cell function, which may provide new therapeutic strategies for cancer. Based on the close relationship between inflammasomes and tumors, targeting inflammasomes is likely to be a key strategy for improving cancer treatment. Inflammation may be targeted in cancer therapy by activating anti-cancer immune cells, such as DCs, NK cells, NKT cells, cytotoxic T cells, Th1 cells and B cells to enhance their anti-tumor activities. At the same time, immunotherapies should aim to suppress cancer-promoting immune cells such as mast cells, TAMs, MDSCs, eosinophils, Th2 cells, Th17 cells, Treg cells and Breg cells, or seek to polarize them into anti-tumor types by targeting key signaling pathways to prevent immunosuppressive effects and cancer progression. Further in-depth research is needed to determine the specific roles of DC subsets, macrophages and Treg cells in the activation of inflammasomes.

Considering the complex role of inflammasomes in cancer, it is necessary to study in more detail the role of different inflammasomes in different tissues, different types of cells and different stages of cancer, and explore the mechanism behind these different therapeutic effects. At the same time, to support more effective, personalized cancer treatment, future studies should seek to determine the best application timeline for each treatment model, in order to more precisely develop and prescribe drugs to individual cancer patients. Translational efforts may focus on small molecule drugs, as small molecule inhibitors that directly target inflammasomes are more potent and cost-effective compared to larger molecular biological agents, affording them greater application prospects due to their low concentration and low toxicity. Already, some drugs that target inflammasomes and their downstream effectors, such as MCC950, OLT1177, anakinra and CY-09, have achieved significant efficacy in the treatment of inflammatory diseases such as osteoarthritis, rheumatoid arthritis and inflammatory bowel disease. However, there are still few studies on the application of such drugs for tumors. Therefore, there is also a need to validate key molecular targets of inflammasome activation to design more selective inhibitors for tumor therapy. In addition, activated inflammasomes regulate tumor immunity by secreting the cytokines IL-1β and IL-18, and have shown good therapeutic effect in preclinical models. However, monotherapy strategies that aim to decrease inflammation are subject to the risk of promoting tumor growth. Because of the heterogeneity and plasticity of the TME, tumors can quickly evolve to evade monoclonal antibodies that target a single immune regulatory factor, leading to drug resistance and even promoting tumor progression. In future, the comprehensive use of high-resolution technology, such as multi-omics analysis and single-cell technology, will assist in the identification of more specific inflammasome targets and the exploration of the cellular and molecular levels of the local treatment response. Detailed data on inflammasome activity will help to translate anti-inflammatory therapies into clinical practice, to better improve the quality of life and prolong the survival of cancer patients. Ideal future oncology treatments may include individualized, multi-drug combinations of anti-inflammasome therapies.

Overall, inflammasomes play a nuanced and central role in regulating the tumor microenvironment, with effects on both tumor and immune cells. Given this, inflammasomes and inflammasome-related molecules are potential drug targets for cancer therapy. However, as inflammasomes partake in diverse and complex effects in response to a plethora of different signals, further research is required to identify effective, safe, and appropriate targets for therapeutic development.

## Author contributions

WH and ZD selected the topic. ZD and LL reviewed all published articles and posters, and wrote the manuscript. HJ and XS provided some revising suggestions on the Discussion section. WH, BL, and HJ revised the manuscript. All authors contributed to the article and approved the submitted version.
